# Comparison of ophthalmic toxicity of light-emitting diode and organic light-emitting diode light sources

**DOI:** 10.1038/s41598-020-68565-3

**Published:** 2020-07-14

**Authors:** Ikhyun Jun, Soo Jung Han, Hae-Sol Shin, Jiyeon Kim, Eung Kweon Kim, Tae-im Kim, Sang Chul Yoon, Kyoung Yul Seo

**Affiliations:** 1 Department of Ophthalmology, The Institute of Vision Research, Yonsei University College of Medicine, 50-1 Yonsei-ro, Seodaemun-gu, Seoul, 03722 Republic of Korea; 2 Korea Mouse Sensory Phenotyping Center (KMSPC), Yonsei University College of Medicine, Seoul, Republic of Korea; 3 Brain Korea 21 Plus Project for Medical Science, Yonsei University, Seoul, Republic of Korea; 4 Department of Medical Humanities and Social Sciences, Yonsei University College of Medicine, Seoul, Republic of Korea; 5 Department of Ophthalmology, National Medical Center, 245 Euljiro, Jung-gu, Seoul, 04564 Republic of Korea

**Keywords:** Animal disease models, Eye diseases

## Abstract

The use of organic light-emitting diodes (OLEDs) has rapidly increased in recent years. However, the effect of OLEDs on human health has not been studied yet. We investigated morphologic and functional changes after OLEDs exposure of human ocular cells, including corneal, conjunctival, lens, and retinal pigment epithelial cells, and mouse eyes. In corneal and conjunctival epithelial cells, the levels of reactive oxygen species production and interleukin*-8* expression after white light-emitting diodes (LED) exposure were significantly greater than those after OLED exposure. Although no gross morphologic changes of the eyelid or cornea were found in LED- or OLED-exposed mice, oxidative stress on ocular surface was significantly increased, and the outer nuclear layer (ONL) was significantly shorter in both light-treated groups than the control group. Moreover, ONL thickness was significantly lower in the LED group than the OLED group. The electroretinography response was significantly lower in light exposure group, and there was significant difference between LED- and OLED-treated mice. Although OLED exhibits certain ocular toxicity, it can be less toxic to eyes than LED. The higher blue-wavelength energy of LED light might be the reason for its higher toxicity relative to OLED.

## Introduction

Light-emitting diode (LED) is a semiconductor device that emits light when an electric current is passed through it. Light-emitting-diode lamps are widely used because of their long lifespan and excellent electric efficiency. Recently, LED light sources were introduced in several vitrectomy machines for endo-illumination of the retina^1^. Despite the several advantages of LEDs, many researchers have reported that light from LED sources can cause retinal toxicity^2–5^.

Organic LED (OLED) is an LED containing an organic-compound emissive electroluminescent layer that emits light in response to electric current^6,7^. It is a next-generation light source that can help produce lighter, thinner, and flexible-lighting panels^7^. Because it produces less heat and glare, thus decreasing eye fatigue in users, OLED is considered a human-friendly light source^8^. The use of OLED in lighting and various display equipment is increasing in recent times.

Previous studies have shown that LED light induces oxidative stress and stress-response pathways, which result in retinal cell death^4,9,10^. Both retinal pigment epithelium (RPE) and photoreceptors can be damaged by LED light. Many studies have reported that LED-associated retinal toxicity has wavelength dependency—blue wavelength can induce more functional damage than green or red wavelength^3,4^. Since LED has the best energy efficiency in blue-wavelength light, white LED (WLED) lamps are generally made by combination of a diode that emits short-wavelength radiation and a phosphor that emits a longer wavelength radiation^2^. However, OLED is made by combining diodes that emit three wavelengths of radiation, and, because the energy efficiency of its blue light is not high, the energy of its blue-wavelength light is not as high as that of LED at the same luminance. For this reason, OLEDs might be less toxic to eyes than LEDs. However, OLED-associated toxicity studies have not been conducted yet. Therefore, this study investigated the ophthalmic toxicity of OLED to evaluate the advantages and feasibility of converting from LED to OLED light sources.

## Results

### Reactive oxygen species (ROS) production after light exposure in vitro

**Figure 1:**
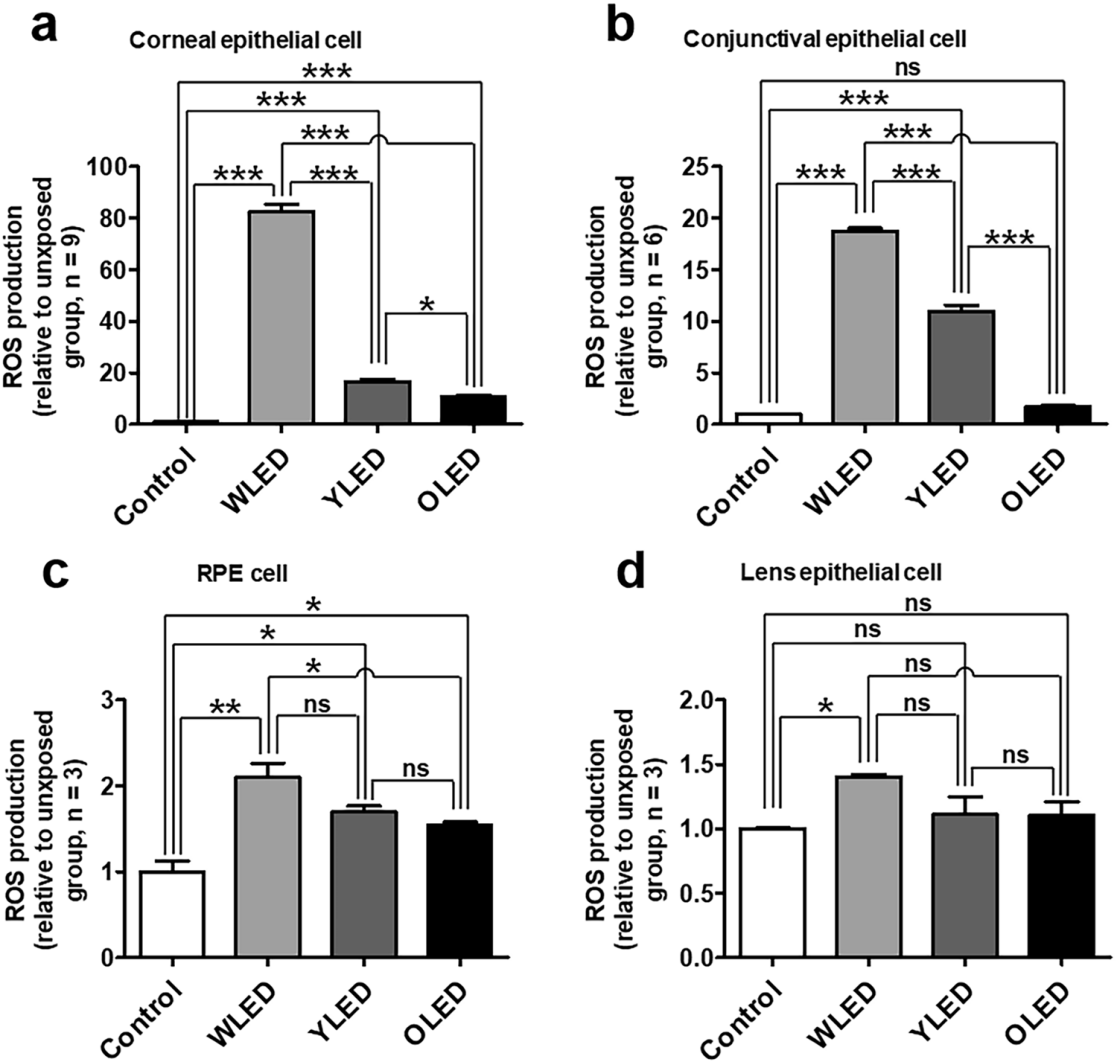
Reactive oxygen species (ROS) production after light exposure in various ophthalmic-tissue-derived cells. ROS production after light exposure in **(a)** corneal epithelial, **(b)** conjunctival epithelial, **(c)** retinal pigment epithelium (RPE), and **(d)** lens epithelial cells. Data are presented as mean ± standard error of mean. ns, not significant; *P < 0.05; **P < 0.01; ***P < 0.001.

In SV40-immortalized human corneal epithelial cells, WLED-exposed cells exhibited significantly higher ROS production (approximately 80-fold higher) than unexposed cells (Fig. 1a, and Supplementary Fig. S1a). Similarly, relative to unexposed cells, Yellow LED (YLED)- and OLED-exposed cells exhibited 17- and tenfold higher levels of ROS production, respectively. There were significant differences in ROS production levels between the WLED and OLED groups. In the Chang cell line, which is derived from conjunctival epithelial cells, ROS production levels were significantly higher in WLED- and YLED-exposed cells (18- and 11-fold higher levels, respectively) than in unexposed cells, although there was no significant difference in this regard between OLED-exposed and unexposed cells (Fig. 1b and Supplementary Fig. S1b). Both ARPE-19 and HLE-B3 cells exhibited similar results as the corneal and conjunctival cell lines (Fig. 1c, d), with ROS production being significantly higher in WLED-exposed cells than in OLED-exposed cells.

### Interleukin *(IL)-8* expression after light exposure in vitro

**Figure 2:**
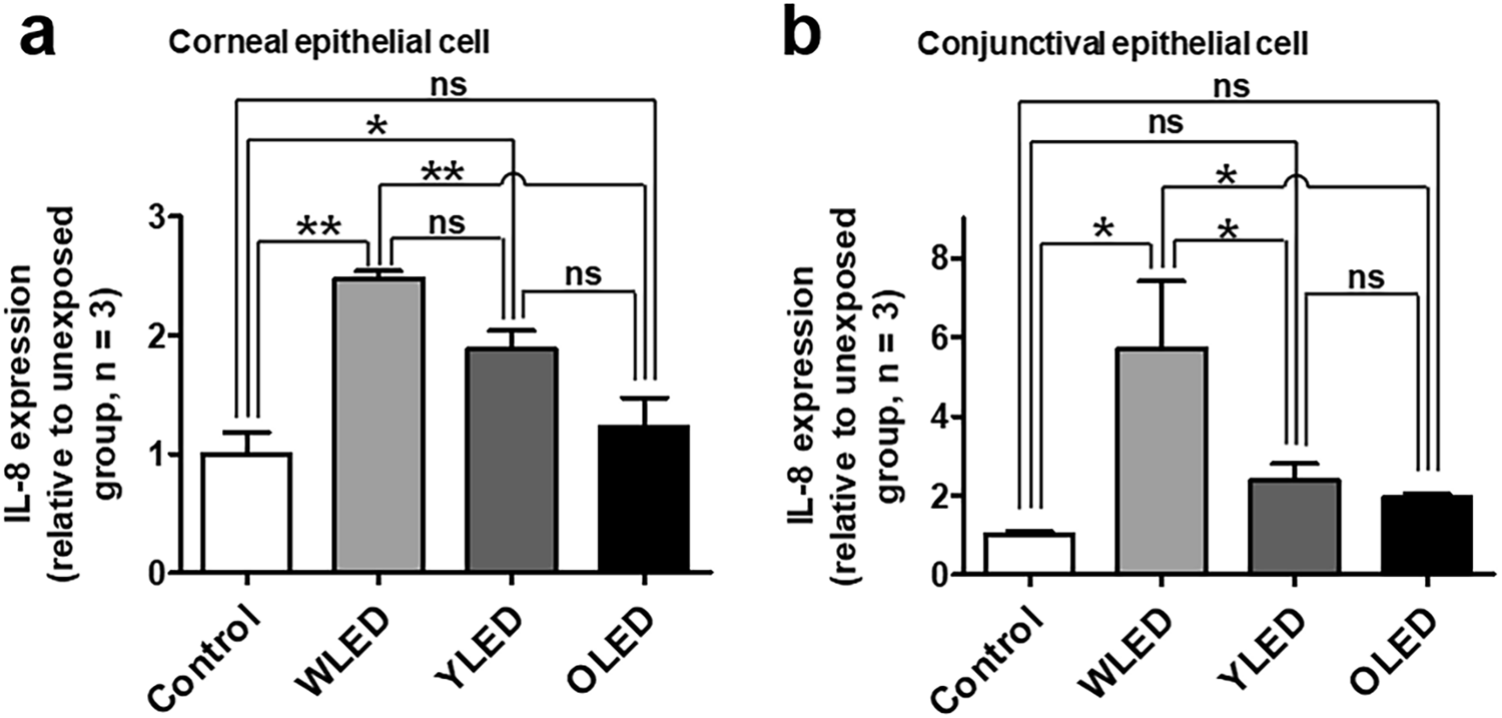
Interleukin (*IL)-8* production after light exposure in various ophthalmic-tissue-derived cells. *IL-8* production after light exposure in **(a)** corneal and **(b)** conjunctival epithelial cells. Data are presented as mean ± standard error of mean. ns, not significant; *P < 0.05; **P < 0.01.

In corneal epithelial cells, LED-exposed cells exhibited significantly higher *IL-8* expression levels than unexposed cells (Fig. 2a); however, there was no significant difference in this regard between OLED-exposed and unexposed cells. In conjunctival epithelial cells, *IL-8* expression levels in WLED-exposed cells were approximately sixfold higher than those in unexposed cells; however, there was no such difference in *IL-8* expression between YLED- or OLED-exposed cells and unexposed cells (Fig. 2b).

### Morphologic and histologic alteration of the anterior segment after light exposure

**Figure 3:**
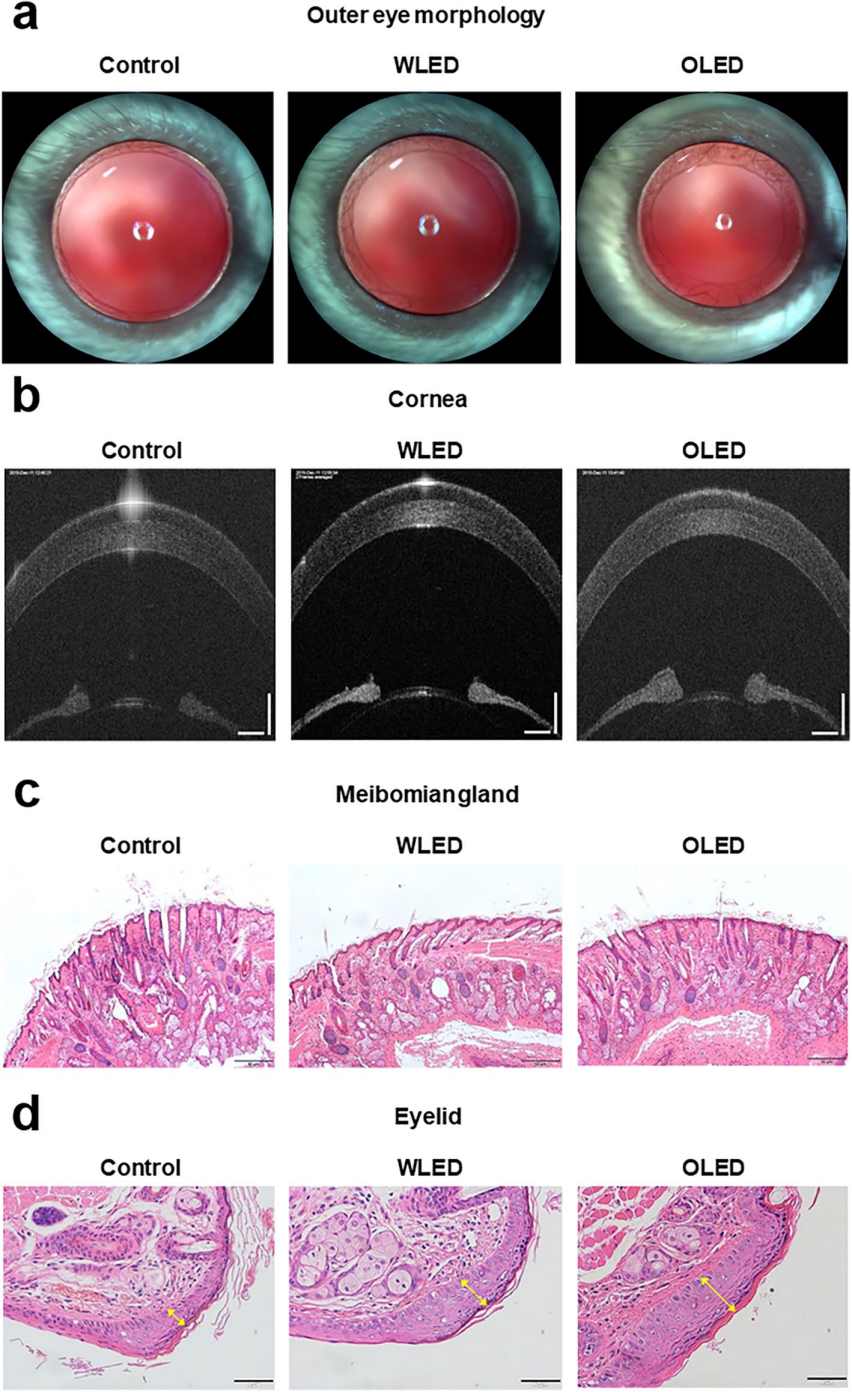
Morphologic and histologic findings of the anterior segment after light exposure. **(a)** Outer-eye morphology after light exposure. **(b)** Optical coherence tomography image of the cornea after light exposure. Histologic findings of **(c)** meibomian glands and **(d)** eyelids after light exposure.

To determine the effect of LED toxicity on the anterior segment of eyes in vivo, we investigated the gross morphology and histologic characteristics of the eyelid and cornea. No definite gross morphologic change of the eyelid or cornea was seen in the LED- and OLED-exposure groups (Fig. 3a). There was no difference in OCT findings of the cornea between the LED- and OLED-exposure groups (Fig. 3b). Furthermore, with regard to optical coherence tomography (OCT) findings of the cornea, there was no difference in either experimental group relative to control mice. In the slit-lamp examination that was performed to evaluate lens opacity after light exposure, no abnormalities were noted in the light-exposure groups (Supplementary Fig. S2). The results of histologic evaluation of the eyelid after LED or OLED exposure revealed no difference in the size or shape of meibomian glands (Fig. 3c); additionally, there was no difference in this regard between the experimental and control groups. The results of histologic evaluation of the eyelid revealed an interesting finding: The epidermis was thicker in both the LED- and OLED-exposure groups than in the control group; however, there was no infiltration of inflammatory cells (Fig. 3d).

### Oxidative stress induced lipid peroxidation on the ocular surface increased after light exposure

**Figure 4:**
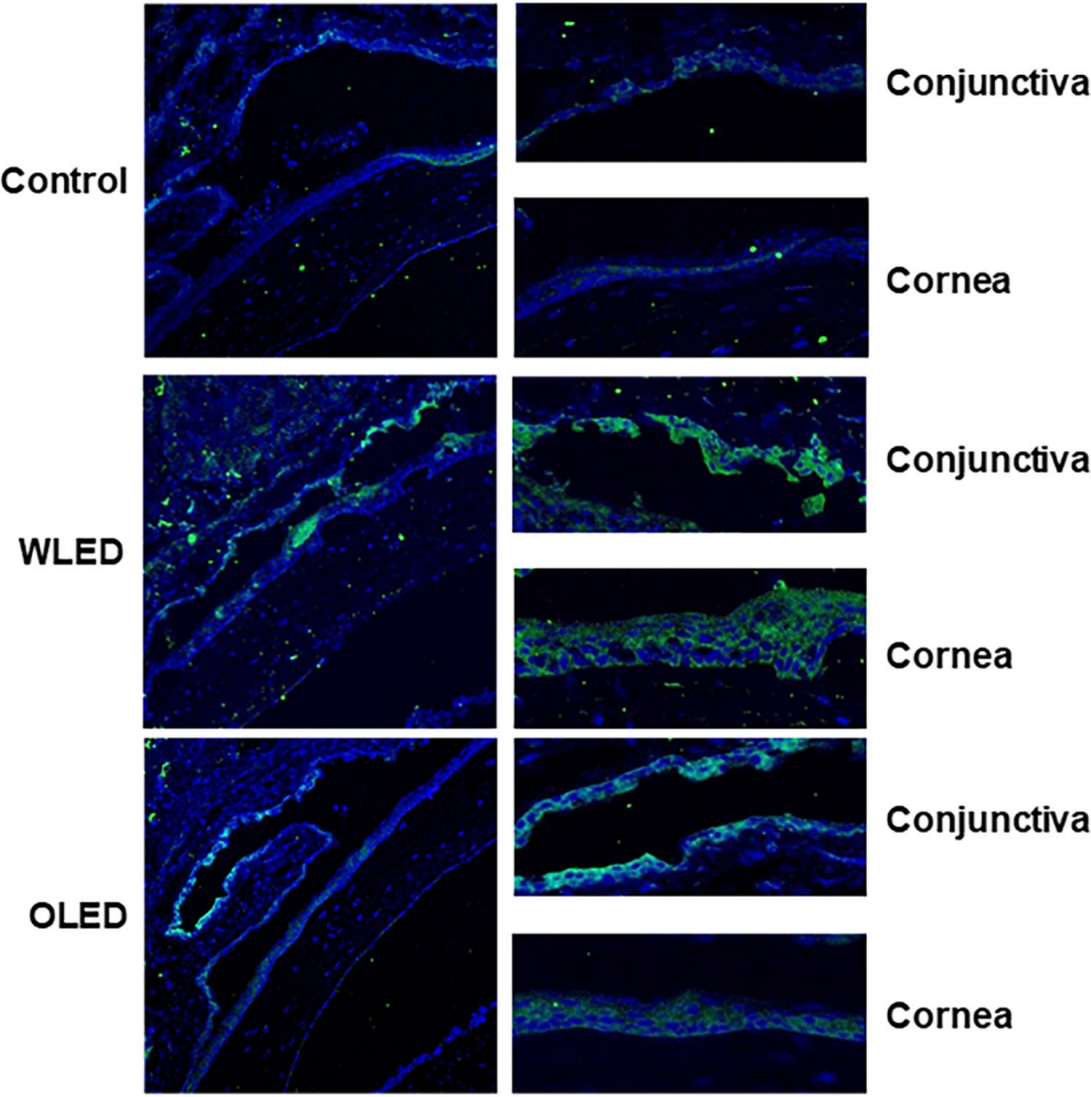
Immunoflourescence of 4-hydroxynonenal (4-HNE), which is a biomarker for oxidative stress of conjunctiva and cornea after 2000 lx light exposure. The intensity of fluorescence was the highest in the LED group, followed by the OLED group, and fluorescence was hardly visible in the control group.

Although ROS production, and IL-8 expression were increased in corneal and conjunctival epithelial cells after light exposure, no significant changes in gross morphology and histology of the ocular surface were observed after LED or LED exposure in the mice. Therefore, we investigated the expression of 4-hydroxynonenal (4-HNE), an oxidative stress induced lipid peroxidation marker, after light exposure, using immunofluorescence to verify molecular level differences (Fig. 4). The expression of 4-HNE was highest in LED exposed mice, followed by OLED exposed mice, and control mice, which was consistent with the results of in vitro experiments.

### Morphologic changes of the retina after light exposure

**Figure 5:**
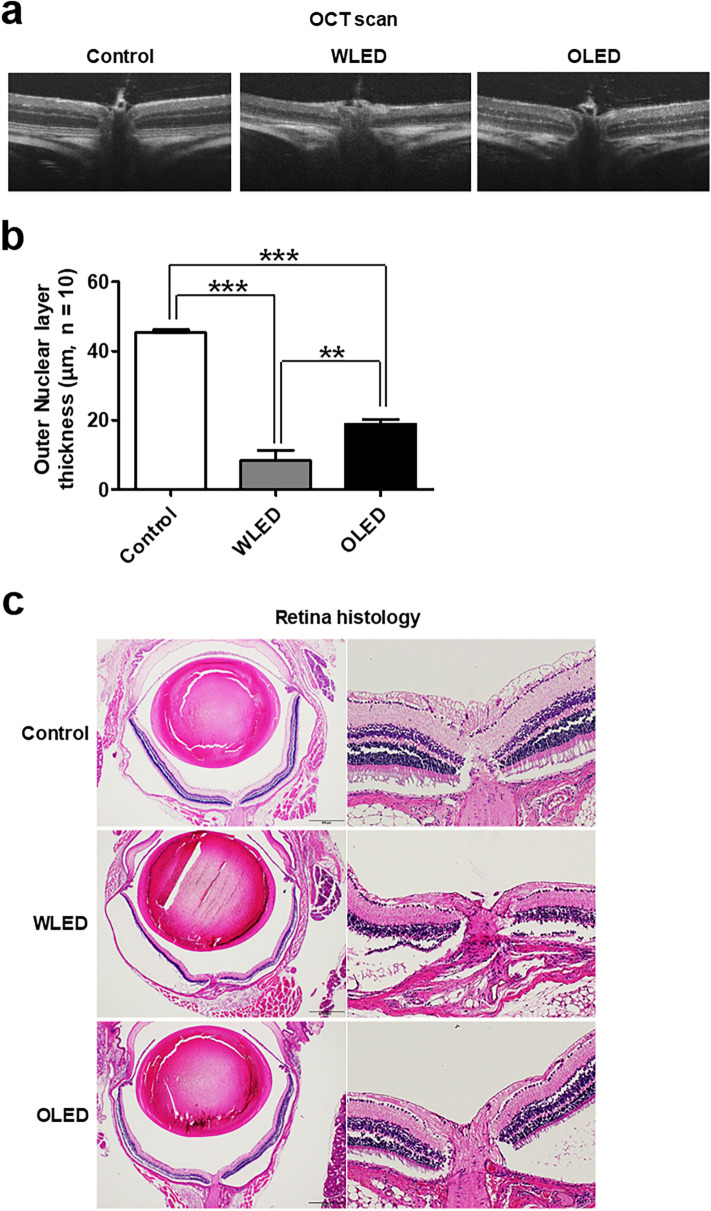
Morphologic and histologic findings of the retina after 2000 lx light exposure. **(a)** Representative optical coherence tomography (OCT) image of the retina after 2000 lx light exposure. **(b)** The retinal thickness of each layer was investigated at a distance of 200 μm from the disc margin and compared among the study groups. There were significant differences in outer nuclear layer (ONL) thickness among the groups. **(c)** The findings of histologic evaluation of the retina after 2000 lx light exposure revealed that the ONL thickness of the light-exposure groups was significantly lower than that of the control group. Data are presented as mean ± standard error of mean. **P < 0.01; ***P < 0.001.

The results of fundus photography for investigating retinal gross morphology and vessel configurations (Supplementary Fig. 3a) revealed no abnormal findings in LED- or OLED-exposed mice relative to control mice. Since several previous reports have reported a decrease in the number of photoreceptor rows and thickness of the outer nuclear layer (ONL) after light exposure^3,11–13^, we measured ONL thickness by OCT. First, we conducted the experiments using 1,000 lx illuminance lights to imitate the brightness used in general household-lighting conditions. Relative to the control group, the LED- and OLED-exposure groups both exhibited significantly lesser ONL thickness; however, ONL thickness was significantly greater in the OLED-exposure group than in the LED-exposure group (Supplementary Fig. 3b). The same tendency was noted upon histopathologic evaluation of the retina (Supplementary Fig. 3c). However, since the difference between the LED and OLED groups was not clearly visible, we conducted additional experiments using stronger light (2000 lx). A more apparent difference in ONL thickness was found between the three groups (Fig. 5a). The ONL thickness was 45.33 ± 2.07, 8.40 ± 9.31, and 18.89 ± 4.31 μm in the control, LED exposure, and OLED exposure groups, respectively (Fig. 5b). Along with the OCT results, the retinal histology results showed that the ONL thickness was thinnest in the LED exposure group, and the OLED exposure group showed considerably thinner results (Fig. 5c).

### Electroretinography (ERG) measurements after light exposure

**Figure 6:**
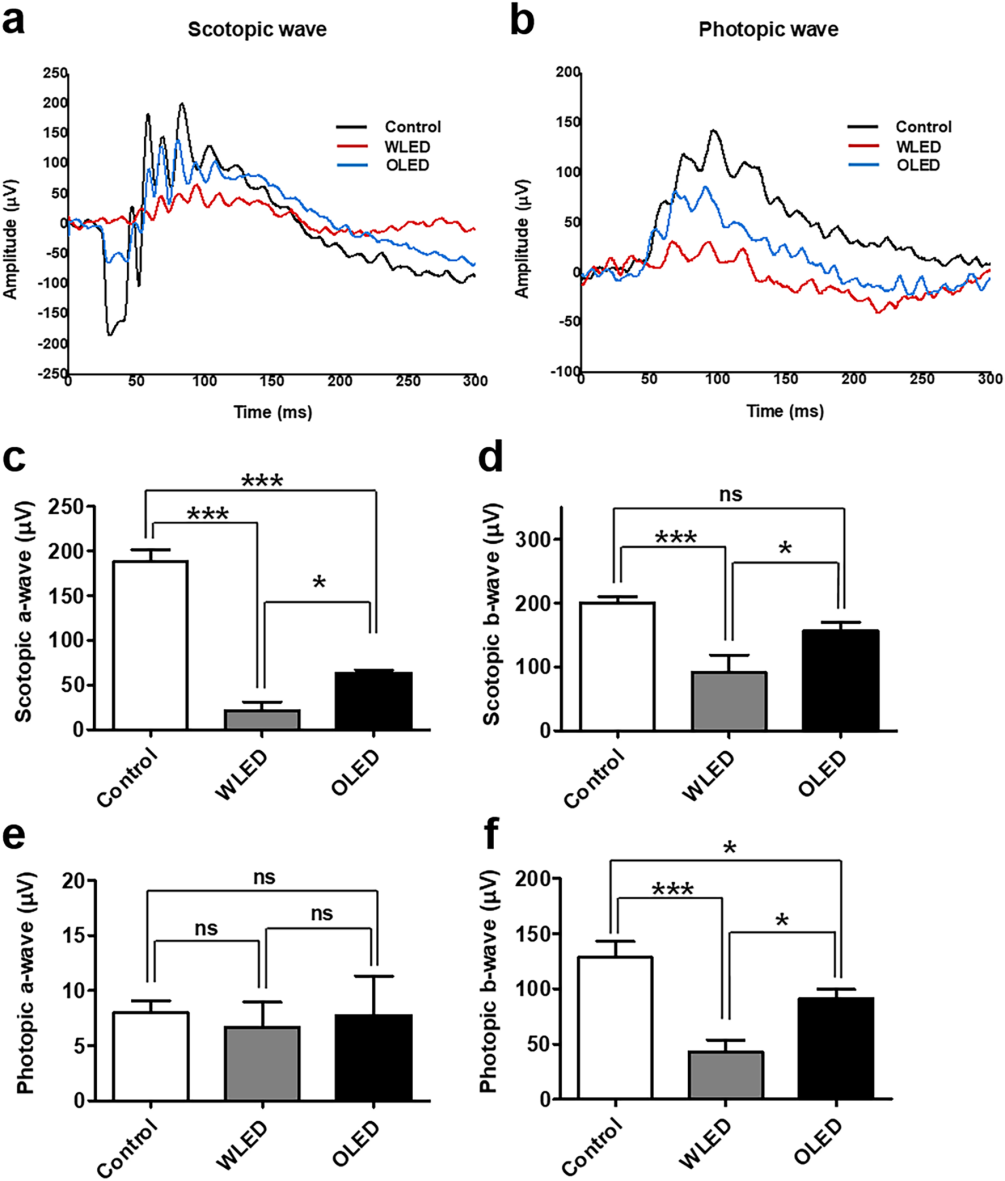
Electroretinogram (ERG) findings after 2000 lx light exposure. Representative traces of **(a)** scotopic and **(b)** photopic ERG after 2000 lx light exposure. Mean amplitudes of scotopic a **(c)** and b **(d)** waves and photopic a **(e)** and b **(f)** waves after light exposure are summarized. Data are presented as mean ± standard error of mean. ns, not significant; *P < 0.05; ***P < 0.001.

According to previous studies, ERG response can be attenuated by LED exposure^9,14^. Therefore, we measured ERG responses in mice after light exposure. In 1,000 lx illuminance experiments (Supplementary Fig. 4), the amplitude of ERG response was significantly lower in the light exposure groups than the control group in scotopic a wave, b wave, and photopic b wave. However, there was no significant statistical difference between the LED and OLED groups. In contrast, in 2000 lx intensity experiments, significant differences between groups were noted (Fig. 6). Representative ERG response traces of 2000 lx LED- and OLED-exposed mice are shown in Fig. 6. The amplitudes of the scotopic a wave, and photopic b wave were significantly lower in LED- and OLED-exposed mice than in control mice (Fig. 6c). The amplitudes of scotopic b waves after LED exposure were significantly lower compared to those in the control group; in contrast, while the corresponding amplitudes after OLED exposure tended to be lower compared to those in the control group, the differences were not statistically significant (Fig. 6d ). Furthermore, the amplitudes of scotopic a, and b waves, and photopic b waves after LED exposure were significantly lower than the OLED exposure group.

## Discussion

We investigated the phototoxicity of OLED light on eyes in this study. Overall, our findings indicate that OLED is less toxic than LED, although even OLED might be toxic to eyes.

Because they provide energy-efficient lighting, LEDs are being increasingly positioned as alternative light sources in the lighting market. However, LED-associated phototoxicity in eyes has become an issue. The phototoxicity of LEDs has been studied mainly in the retina, where it causes oxidative stress in photoreceptors and the RPE, resulting in activation of stress-response and cell-death pathways^4,9–11^. Exposure to LEDs can cause damage to not only the retina but also the ocular surface^15,16^. Overexposure to blue-wavelength LED light attenuates the viability of corneal epithelial cells and increases ROS production and inflammation in the ocular surface of mice^15,16^.

Although the effect of LED on eyes has been extensively studied, the phototoxicity of OLED on eyes has not yet been evaluated. Many OLED display and indoor-lighting devices are being used because they allow flexibility for design while providing clear images. Owing to these advantages, the market for OLED panels for mobile devices and OLED indoor lighting has been gradually expanding.

Similar to previous findings, our results showed that ROS production in various ophthalmic-tissue-derived cells was significantly higher in the LED-exposure group than in the control group (Fig. 1). Although ROS production was also higher in the OLED-exposure group than in the control group, the difference was much lower than that observed in case of WLED-exposed cells. It is well known that LED toxicity is mainly due to its blue-wavelength light^3,11,12^; it has been found that, the lower the correlated color temperature, the lesser the toxicity^10^. Because combination of complementary wavelengths creates a white-light sensation on the human eye, WLED is commonly manufactured by combining a diode emitting a short-wavelength light with a phosphor emitting a longer wavelength light^2^. As shown in Supplementary Figure S5, the energy of the blue wavelength of LED light is the highest; hence, the toxicity of LED light might be higher than that of OLED light, which has three different peaks of energy. Therefore, we measured ROS production after exposure to YLED—which has a similar correlated color temperature as OLED—and found that ROS production levels after YLED exposure were lower than those after WLED exposure and higher than those after OLED exposure. The relatively low ROS production level after YLED exposure can be explained by the lower energy of blue-wavelength light in YLED compared to that in WLED. However, ROS production levels in the YLED group were still higher than those in the OLED group, which might be associated with the WLED-like of the spectral graph and the high-energy peak of the blue wavelength of YLED light. While LED light produces two peaks (blue and yellow) on the spectral graph, OLED light produces three (red, green, and blue), which means that YLED light requires relatively high energy in the blue wavelength to produce the same correlated color temperature as OLED.

In primary human RPE cells, LED illumination upregulates vascular endothelial growth factor-A, *IL-6*, and *IL-8* expression and downregulates monocyte chemoattractant protein-1 expression; these changes are associated with mitogen-activated protein kinase and nuclear factor-κB signaling pathways^10^. Inflammatory cytokines, including *IL-1, IL-6, IL-8*, and tumor necrosis factor-α, are upregulated by oxidative stress after exposure of the ocular surface to ultraviolet (UV)-B radiation^17^. Lee et al.^15^ reported a significant increase in *IL-1β* and *IL-6* expression after blue-LED exposure of the ocular surface in mice; however, the authors did not evaluate changes in *IL-8* expression. Although *IL-8* is not usually produced in normal cornea, it can be detected in several pathological conditions, including dry eyes, atopic keratoconjunctivitis, herpes keratitis, and fungal infection^18–20^. Oxidative stress is associated with inflammation of the cornea and conjunctiva, which may induce dry eyes, keratoconus, and Fuchs’ endothelial dystrophy^21^. Specifically, *IL-8* is one of the proinflammatory cytokines associated with oxidative stress, which is known to play an important role in dry eyes^22,23^. Therefore, we measured *IL-8* expression levels after light exposure and found significantly higher expression levels in corneal and conjunctival epithelial cells exposed to LED irradiation than in unexposed cells (Fig. 2). On the other hand, OLED exposure did not affect *IL-8* expression levels in corneal and conjunctival epithelial cells. These results can be understood in the same context as the findings of the ROS production experiments. Similar to the results of in vitro experiments, we could confirm that the oxidative stress of the ocular surface increased in vivo. It suggests that light exposure which increase the oxidative stress on the ocular surface, can be a risk factor for dry eye or ocular surface inflammatory diseases.

The thickness of the ONL is known to decrease after LED exposure in a time-, intensity-, and wavelength-dependent manner^3,4,11,12^. In the present study, also, the ONL thickness was significantly lesser in LED-exposed mice than in control mice. Moreover, the ONL thickness in OLED-exposed mice was significantly lesser than that in control mice but significantly greater than that in LED-exposed mice (Fig. 5). In line with OCT and retinal-histology data, the amplitudes of the scotopic a waves and photopic b waves were considerably lower in LED- and OLED-exposed mice than in control mice. However, the amplitudes of scotopic a, and b waves, and photopic b waves were significantly lower in the LED group than the OLED group. These findings once again show that OLED might cause less toxicity than LED. In 1,000 lx light treated mice, no abnormal findings in retinal morphology or vessel configuration were noted. These results differ from those of previous studies that reported significant histologic changes after light exposure^9,11^. Most previous studies involved extreme light exposure to high-brightness lighting systems, and few studies have employed general household-lighting conditions^12,24^. This might be a reason for the minimal changes observed in our experiments. The results that the ONL thickness is significantly shorter and the ERG response is prominently affected under 2000 lx experimental condition, support this hypothesis. A previous study reported that pigmented rats are more resistant to light toxicity than albino rats^12^, hence it may be less toxic in pigmented mice and human compared to the non-pigment mice used in this study.

Although the results of overall anterior-segment examination showed no gross abnormalities, an interesting finding was noted in experimental mice, which was that the epidermal thickness of the eyelid was greater in the light-exposed groups than in the control group (Fig. 3). Repeated UV exposure is known to induce significant thickening of the epidermis, which is believed to be a protective reaction by the skin against additional UV-associated damage^25–27^. However, LED is widely used in treatment of skin problems, including acne vulgaris, herpes simplex, herpes zoster, and skin rejuvenation through photobiomodulation^28,29^. Light-emitting-diode treatment on skin might cause several adverse events, including pigmentary changes, erythema, desquamation, dryness, and stinging; however, there is no report on its effect on epidermis thickening^28^. Further mechanistic investigations for this phenomenon are needed.

In conclusion, we propose that, because of its lower blue-wavelength energy, OLED light may be less toxic to eyes than LED light. These results indicate that we might need to switch from LED to OLED lights. In fact, the usage of OLED display devices is rapidly increasing. However, given that OLED light still showed certain ocular toxicity in our study, the threshold limit of OLED light intensity and the detailed mechanistic background of OLED-induced toxicity should be further investigated.

## Methods

### Cell culture

An SV40-immortalized human corneal epithelial cell line was cultured in Dulbecco’s modified Eagle’s medium (DMEM; Hyclone, Logan, UT, USA), and human conjunctival cells (Chang cell line) were cultured in Roswell Park Memorial Institute 1640 medium (Hyclone). Adult retinal pigment epithelial cells (ARPE-19 cells) were cultured in DMEM/Ham’s F12 medium (Invitrogen, Carlsbad, CA, USA), and human lens epithelial cells (HLE-B3 cells) were cultured in minimum essential medium (Hyclone). All media were supplemented with 10% (v/v) fetal bovine serum (Invitrogen), 0.1 mg/mL streptomycin, and 100 U/mL penicillin (Invitrogen). All cells were grown in a humidified incubator at 37 ℃ in 5% CO_2_.

### Animals

This study was approved by the Committee on Animal Research at Yonsei Medical Center. All animal studies were performed in accordance with the Yonsei Medical Center Animal Research Guidelines, which adhere to the standards articulated in “Association for Assessment and Accreditation of Laboratory Animal Care International”(AAALAC) guidelines, and the ARVO Statement for the Use of Animals in Ophthalmic and Vision Research. Eight-week-old female BALB/c mice were purchased from Orient Bio Inc.(Sungnam, Korea).

### Light exposure

This study employed WLED [PBA-0422-04 (5700 K), RFsemi Technologies, Inc., Daejeon, Korea], YLED [PBA-0422-04 (4000 K), RFsemi Technologies, Inc.], and OLED [LL056RS1-54P1 (4000 K), LG Display Co., Ltd., Seoul, Korea] lamps as light sources. The YLED lamp was used for matching color temperature with the OLED lamp. Spectral power distribution of the lamps is presented in Supplementary Figure S5. Cells were exposed to 10,000 lx of light for 45 min at room temperature. Female mice were exposed to 1,000 lx of WLED or OLED light for 12 h per day for 8 weeks (lights on at 08:00 AM; Supplementary Fig. S6) to simulate the brightness of a typical indoor illumination. In addition, 2000 lx of WLED or OLED light was applied to clarify the difference between WLED and OLED. The illuminance of light was continuously evaluated, and maintained according to the conditions throughout the experimental period (Supplementary Fig. S6b). The ambient temperature during light exposure was maintained at 24 ± 2 ℃.

### Measurement of cellular ROS production following light exposure

Intracellular ROS production was estimated using the fluorescent dye 2′,7′-dichlorofluorescein diacetate (DCFDA; Abcam, Cambridge, UK). Cells were stained by incubation with 25 μM DCFDA for 45 min at 37 ∘C and then washed with phosphate-buffered saline. After staining, the cells were exposed to 10,000 lx of light for 45 min at room temperature, following which their fluorescence intensity was measured using a microplate reader (Thermo Fisher Scientific, Vantaa, Finland) at 485 (excitation) and 535 (emission) nm.

### RNA extraction and quantitative real-time polymerase chain reaction (qRT-PCR) analysis

Total RNA was extracted using the TRIzol reagent (Invitrogen) in accordance with the manufacturer's protocol. Complementary DNA was synthesized from total RNA using Moloney Murine Leukemia Virus reverse transcriptase (Thermo Fisher Scientific, Waltham, MA, USA). All qRT-PCR analyses were performed with the SYBR Green real-time PCR master mix (Thermo Scientific, Wilmington, DE, USA) using the ABI 7300 Real-Time instrument (Applied Biosystems, Foster City, CA, USA) as previously described^30^. Expression levels of glyceraldehyde 3-phosphate dehydrogenase (*GAPDH*) and *IL-8* genes were estimated by qRT-PCR using the following primers: *IL-8* forward primer: 5′-CTGGCCGTGGCTCTCTTG-3′; *IL-8* reverse primer: 5′-CCTTGGCAAAACTGCACCTT-3′; *GAPDH* forward primer: 5′-CGGGAAGCTTGTGATCAATGG-3′; and *GAPDH* reverse primer: 5′-GGCAGTGATGGCATGGACTG-3′.

### Anterior and fundus photography, optical coherence tomography (OCT), and electroretinography (ERG)

Prior to investigation, the pupils of mice were dilated with eye drops containing a mixture of 0.5% tropicamide and 0.5% phenylephrine (Mydrin-P, Santen Pharmaceutical Co, Ltd., Osaka, Japan). The mice were then anesthetized by intraperitoneal injection of Xylazine (10 mg/kg; Rompun, Bayer Animal Health, Leverkusen, Germany) and zolazepam–tiletamine (30 mg/kg; Zoletil 50, Vibrac, Carros, France). Anterior, retinal, and OCT images were acquired using the Micron IV imaging microscope (Phoenix Research Labs, Pleasanton, CA, USA). Retinal thickness was measured using the InSight—Animal OCT Segmentation Software (Phoenix Research Labs). The retinal thickness was measured perpendicularly at a distance of 200 μm from the disc margin. In order to make the measured area the same, the horizontally taken image was used (Supplementary Fig. 3). The ERG recordings were obtained using Micron IV technology (Phoenix Research Labs). Subdermal needle electrodes were inserted underneath the skin at the base of the tail (ground electrode) and between the eyes on the forehead (reference electrode). The ERG was recorded as a full-field ERG in accordance with the manufacturer’s standard protocol. Five responses to light stimulation were averaged, and a wave (as a measure of photoreceptor function), b wave (as a measure of bipolar cell function), amplitude, and implicit times of rod and cone responses were determined.

### Histologic analysis

For histologic analysis, the whole eyes of light-exposed mice were harvested, fixed in 4% paraformaldehyde, and processed for routine paraffin embedding and sectioning. Histologic assessment was performed by standard hematoxylin and eosin staining.

### Statistical analysis

The results of multiple experiments are presented as mean ± standard error of mean. Values of *P* < 0.05 were considered significant. Statistical analysis was performed with Student’s t-test or analysis of variance followed by Dunnett’s multiple comparison tests as appropriate.

## Supplementary information

Supplementary Information. (PDF 554 kb)

## Data Availability

The datasets generated during and/or analysed during the current study are available from the corresponding author on reasonable request.
