# Supplementary material for: Comparison of ophthalmic toxicity of light-emitting diode and organic light-emitting diode light sources
Source: Sci Rep. 2020 Jul 14;10:11582. doi: 10.1038/s41598-020-68565-3 (PMC7360575; doi:10.1038/s41598-020-68565-3)
Supplement: Supplementary file 1 — Supplementary Information. (PDF 554 kb) [file 41598_2020_68565_MOESM1_ESM.pdf]

Comparison of Ophthalmic Toxicity of Light-Emitting Diode and Organic Light-Emitting Diode Light Sources

Ikhyun Jun[1,*]; Soo Jung Han[1,*]; Hae-Sol Shin[1,2], Jiyeon Kim[1,2], Eung Kweon Kim[1]; Tae-im Kim[1]; Sang Chul Yoon[1,3,**]; Kyoung Yul Seo[1,2,**]

[1] The Institute of Vision Research, Department of Ophthalmology, Yonsei University College of Medicine, Seoul, Republic of Korea

[2] Brain Korea 21 Plus Project for Medical Sciences, Yonsei University College of Medicine, Seoul, Republic of Korea

[3] Department of Medical Humanities and Social Sciences, Yonsei University College of Medicine, Seoul, Republic of Korea

Supplementary Figure S1.

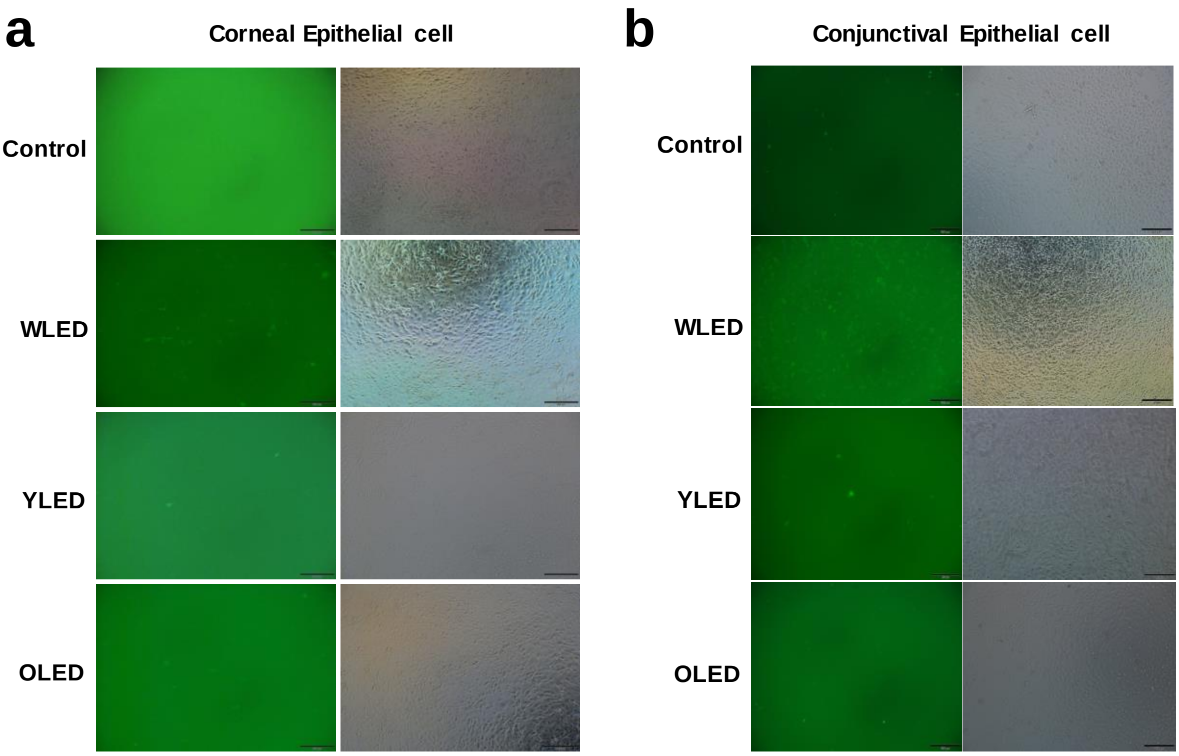

Supplementary Figure S1. Reactive oxygen species (ROS) generation after light exposure was estimated using the DCFDA fluorescent dye in corneal epithelial cells **(a)**, and conjunctival epithelial cells **(b)**. The fluorescence intensity of LED exposed cells was highest, followed by YLED and OLED.

Supplementary Figure S2.

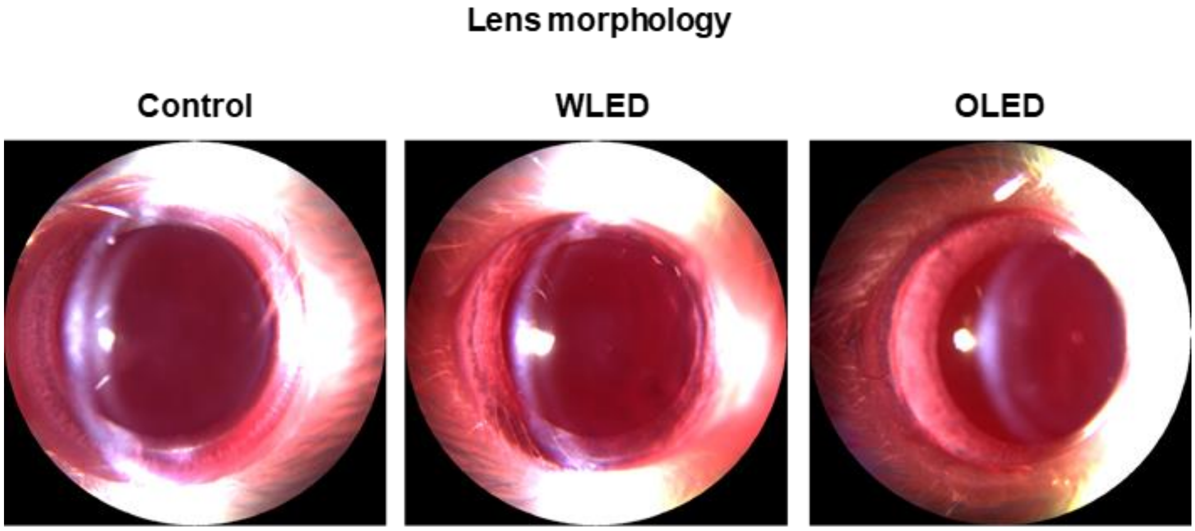

Supplementary Figure S2. Slit-lamp examination of crystalline lens after light exposure. No abnormality was noticed after light exposure.

Supplementary Figure S3.

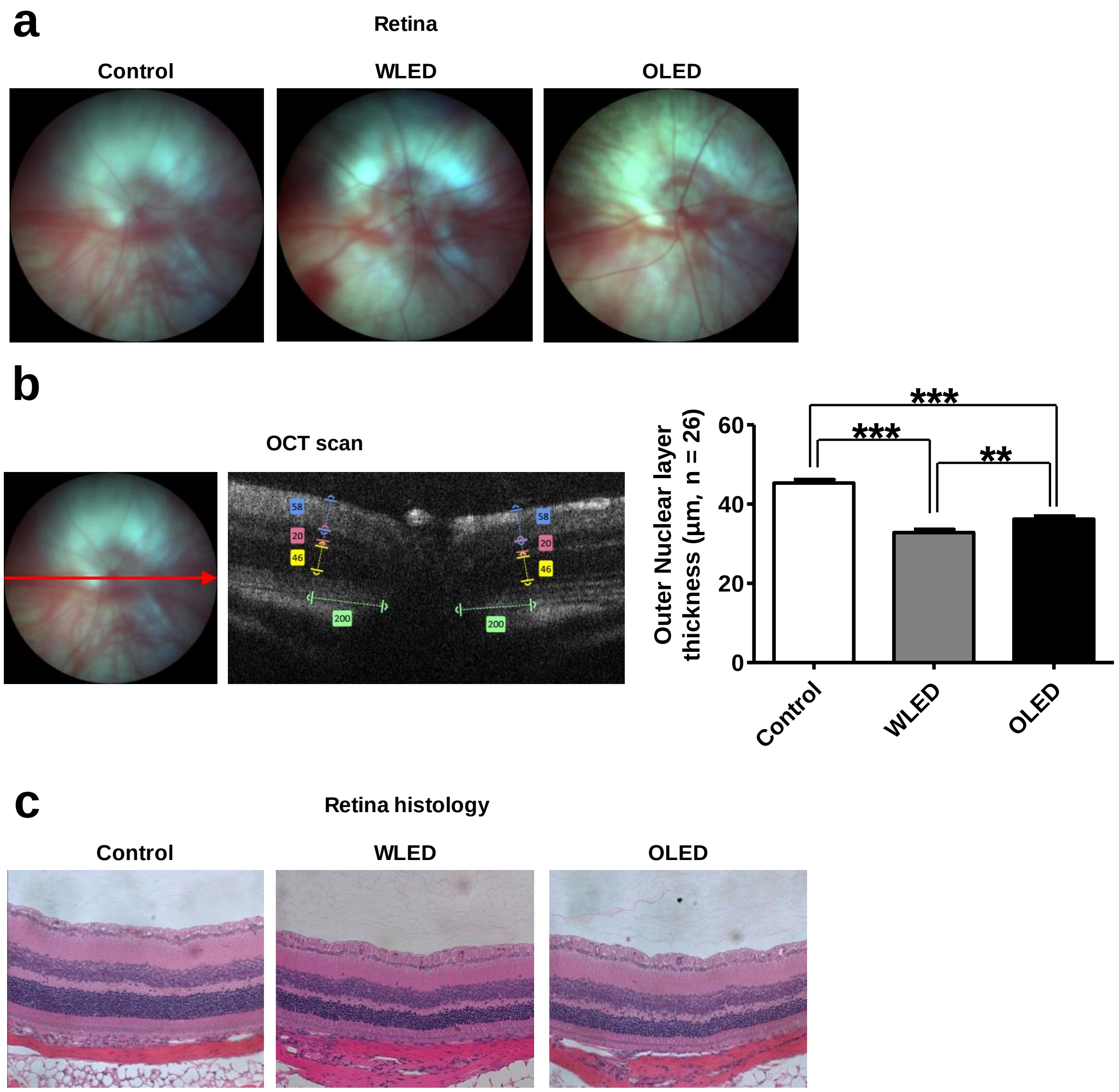

Supplementary Figure S3. Morphologic and histologic findings of the retina after 1000 lux light exposure. (a) Fundus photography after light exposure (b) Optical coherence tomography (OCT) image of the retina after light exposure. The thickness of each layer was investigated at a distance of 200 μm from the disc margin and compared among the study groups. There were significant differences in outer nuclear layer (ONL) thickness among the groups after 1000 lux light exposure, however the differences between groups were smaller than those after 2000 lux light exposure. (c) The findings of histologic evaluation of the retina after light exposure revealed that the ONL thickness of the light-exposure groups was significantly lower than that of the control group. Data are presented as mean ± standard error of mean. **$P < 0.01$; ***$P < 0.001$.

Supplementary Figure S4.

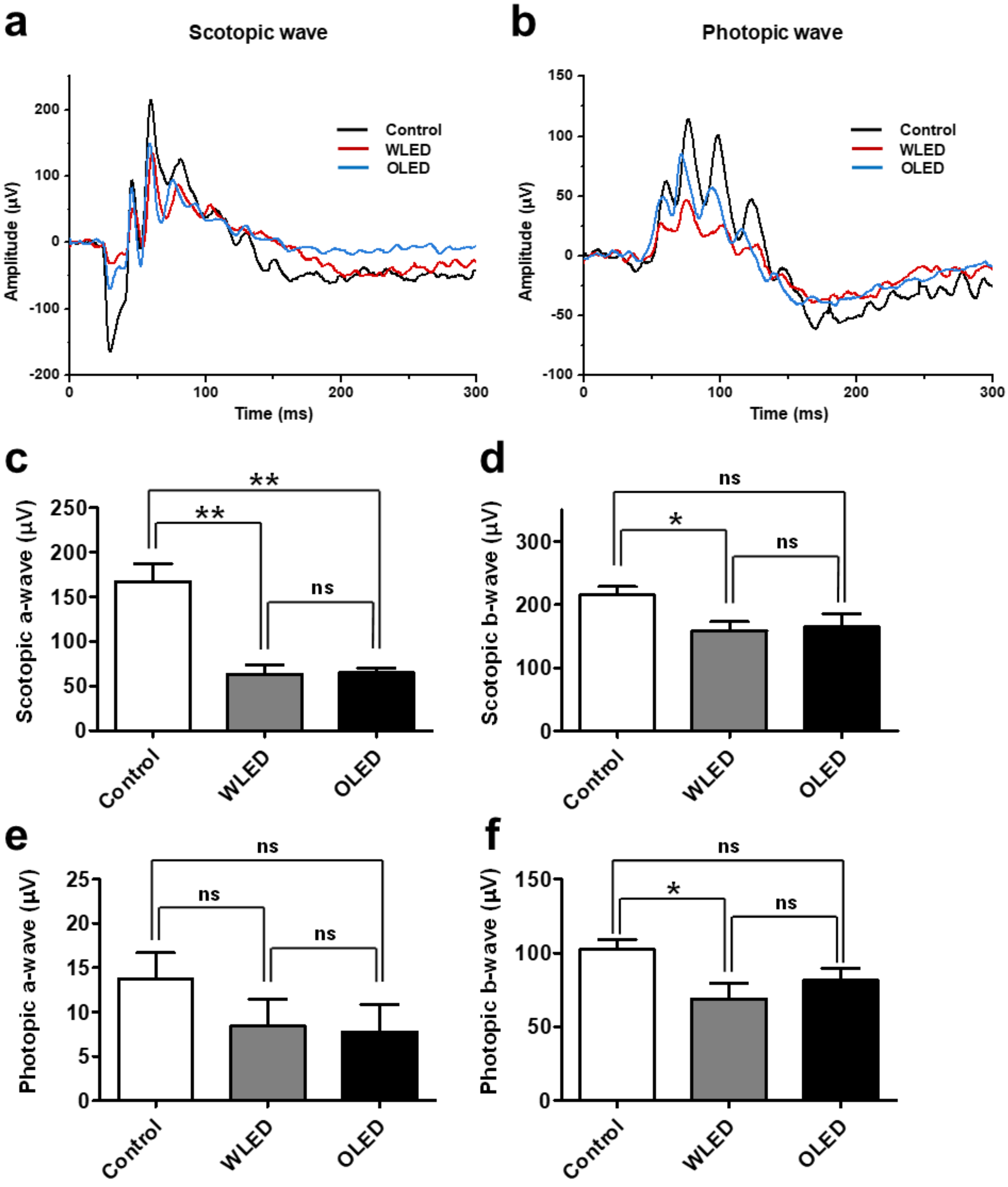

Supplementary Figure S4. Electroretinogram (ERG) findings after 1000 lux light exposure. Representative traces of **(a)** scotopic and **(b)** photopic ERG after light exposure. Mean amplitudes of scotopic a **(c)** and b **(d)** waves and photopic a **(e)** and b **(f)** waves after light exposure are summarized. Data are presented as mean ± standard error of mean. ns, not significant; **P* < 0.05; ***P* < 0.01.

Supplementary Figure S5.

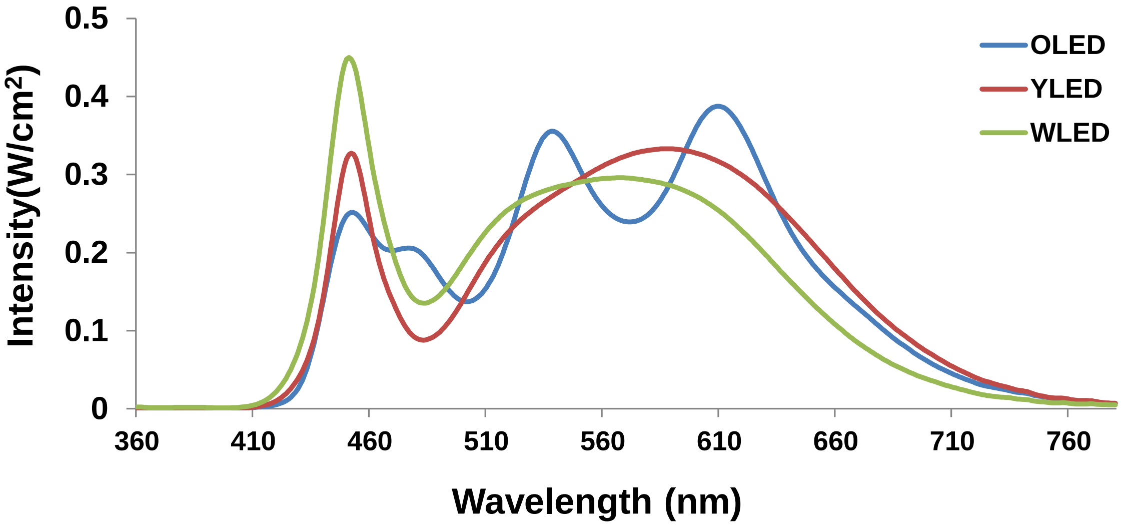

Supplementary Figure S5. Spectral power distribution curves of the light sources.

Supplementary Figure S6.

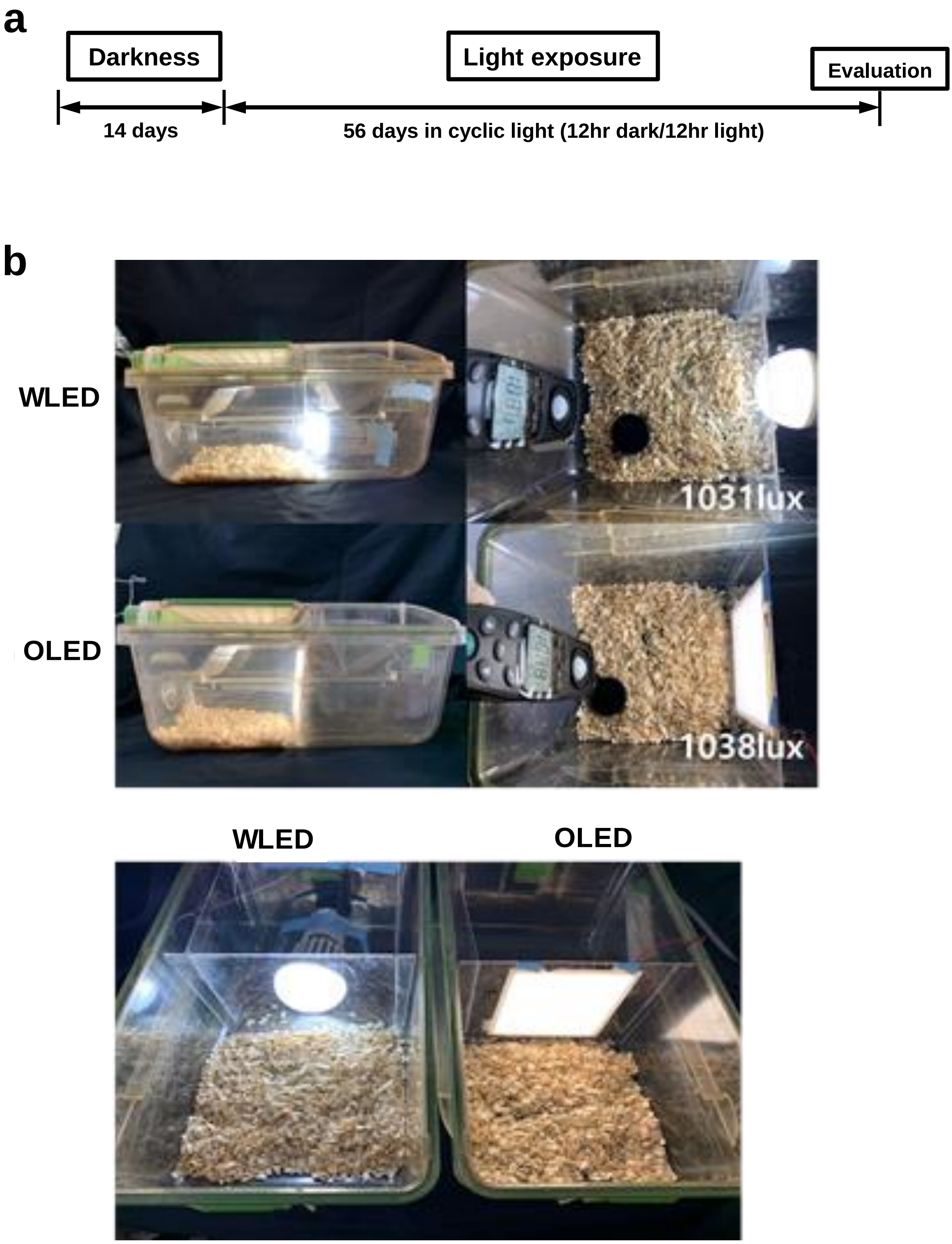

Supplementary Figure S6. Schematic process of *in vivo* light exposure. **(a)** Time schedule of *in vivo* light exposure experiment. **(b)** The appearance of *in vivo* light exposure experiment.
